# Induction of complement proteins in a mouse model for cerebral microvascular Aβ deposition

**DOI:** 10.1186/1742-2094-4-22

**Published:** 2007-09-18

**Authors:** Rong Fan, Kelly DeFilippis, William E Van Nostrand

**Affiliations:** 1 Department of Medicine, Stony Brook University, Stony Brook, NY 11794 USA

## Abstract

The deposition of amyloid β-protein (Aβ) in cerebral vasculature, known as cerebral amyloid angiopathy (CAA), is a common pathological feature of Alzheimer's disease and related disorders. In familial forms of CAA single mutations in the Aβ peptide have been linked to the increase of vascular Aβ deposits accompanied by a strong localized activation of glial cells and elevated expression of neuroinflammatory mediators including complement proteins. We have developed human amyloid-β precursor protein transgenic mice harboring two CAA Aβ mutations (Dutch E693Q and Iowa D694N) that mimic the prevalent cerebral microvascular Aβ deposition observed in those patients, and the Swedish mutations (K670N/M671L) to increase Aβ production. In these Tg-SwDI mice, we have reported predominant fibrillar Aβ along microvessels in the thalamic region and diffuse plaques in cortical region. Concurrently, activated microglia and reactive astrocytes have been detected primarily in association with fibrillar cerebral microvascular Aβ in this model. Here we show that three native complement components in classical and alternative complement pathways, C1q, C3, and C4, are elevated in Tg-SwDI mice in regions rich in fibrillar microvascular Aβ. Immunohistochemical staining of all three proteins was increased in thalamus, hippocampus, and subiculum, but not frontal cortex. Western blot analysis showed significant increases of all three proteins in the thalamic region (with hippocampus) as well as the cortical region, except C3 that was below detection level in cortex. Also, in the thalamic region (with hippocampus), C1q and C3 mRNAs were significantly up-regulated. These complement proteins appeared to be expressed largely by activated microglial cells associated with the fibrillar microvascular Aβ deposits. Our findings demonstrate that Tg-SwDI mice exhibit elevated complement protein expression in response to fibrillar vascular Aβ deposition that is observed in patients with familial CAA.

## Background

Abnormal accumulation of amyloid β peptides (Aβ) in brain is one of the major pathological characterizations of Alzheimer's disease (AD) and related disorders [1]. Aβ, a 39–42 residue proteolytic product of the amyloid-β peptide precursor (AβPP) by β- and γ-secretase cleavages, possesses a high propensity to self-assemble into β sheet-rich fibrils [2]. One prominent site of brain Aβ deposition is in and along the walls of cerebral blood vessels, a condition known as cerebral amyloid angiopathy (CAA), which is frequently found in AD [3,4]. In contrast to parenchymal plaques, which can be composed of either diffuse or fibrillar deposits, cerebral vascular Aβ deposits appear to be exclusively fibrillar in nature [5]. Several familial forms of CAA result from specific point mutations within the mid-region of the Aβ domain, which significantly enhance the fibrillogenic and cerebral vascular cytotoxic properties of Aβ [6-8].

Previously, we generated transgenic mice that express neuronally derived human AβPP, containing the Dutch-type (E693Q) and Iowa-type (D694N) familial CAA mutations, the Tg-SwDI mice [9]. These mice were shown to develop early-onset and robust deposition of cerebral microvascular fibrillar amyloid but only diffuse parenchymal Aβ plaques. The microvascular accumulation of fibrillar Aβ is most prominent in the thalamic and subiculum regions of the brain [9]. In these areas with the most fibrillar microvascular Aβ deposits, activated inflammatory cells, microglia and astrocytes, were also found highly enhanced [10]. Several pro-inflammatory cytokines were shown to be elevated in Tg-SwDI mouse brains, indicating the active synthesis of inflammatory molecules by these cells [10,11].

Another important aspect of the neuroinflammatory response in AD and CAA is the innate immune system activation, among which, complement is a key player [12]. Complement proteins have been shown to be induced and associated with Aβ plaques in brains of AD patients and familial CAA patients as well as AD animal models, specifically those plaques containing the fibrillar form of the Aβ peptide [13-15]. The overall outcome of complement activation depends on the balance of its detrimental and beneficial effects [16]. On one side, complement activation could induce cell lysis and cause cell death [17,18]; on the other side, complement components such as C1q and C3b can promote the clearance of cellular debris and apoptotic cells and enhance cell survival [15,19]. Although complement proteins are typically secreted by immune cells, in the CNS, microglia, astrocytes, and neurons have been reported being capable of producing complement components upon stimulation [20,21]. In the current study, we investigated the expression of several native complement components in twelve months old Tg-SwDI and wild-type C57BL/6 mice, and found they were increased significantly in association with microvascular amyloid deposits and co-localized with activated microglial cells. The finding that microglial synthesis of the native complement proteins was induced suggests that early complement activation may be increased in the Tg-SwDI mouse model of cerebral microvacular Aβ deposition.

## Methods

### Animals

Generation of Tg-SwDI transgenic mice on a pure C57BL/6 background was recently described [9]. These mice express low levels of human Swedish/Dutch/Iowa mutant AβPP in neurons under control of the mouse Thy1.2 promoter. The deposition of mutant Dutch/Iowa Aβ peptide starts around 3 months of age and begins to plateau at about 15 months in transgenic mice with low variation between individuals. Homozygous Tg-SwDI and nontransgenic C57BL/6 mice at 12 months of age were used in this study. All work with animals followed National Institutes of Health guidelines and was approved by Stony Brook University Institutional Animal Care and Use Committee.

### Histology

At least 24 mice per genotype were euthanized at 12 months of age. After cold-phosphate buffer saline (PBS, 0.1M, pH 7.6) perfusion, brains were removed and dissected through the mid-sagittal plane. One cerebral hemisphere was immersion-fixed with 4% paraformaldehyde overnight at 4°C and subjected to increasing concentrations (10, 20, and 30%) of sucrose in PBS, then embedded in OCT compound (Sakura Finetek Inc., Torrance, CA) and snap-frozen in dry ice. Coronal sections were cut at 20-μm thickness using a Leica CM1900 cryostat (Leica Microsystems, Inc., Bannockburn, IL), and then stored in PBS with 0.02% NaN_3 _at 4°C.

### Immunohistochemistry

Immunostainings were performed on sections mounted on Colorfrost/Plus slides (Fisher Scientific, Houston, TX). Antigen retrieval was performed in 1:100 antigen-unmasking solution (Vector Lab, Burlingame, CA) for 30 min at 90°C for C1q and microglia immunostaining. Nonspecific binding was prevented by incubating sections in blocking buffer (PBS containing 0.1% Triton X-100 and 2% bovine serum albumin (Sigma, St. Louis, MO) for 20 min at room temperature. Brain sections were incubated with primary antibodies diluted in blocking buffer overnight at 4°C. The following primary antibodies were used for immunostaining: mouse monoclonal antibody to glial fibrillary acidic protein (GFAP) for identification of astrocytes (1:300, Millipore, Temecula, CA); mouse monoclonal antibody 5D4 to keratan sulfate for identification of activated microglia (1:300; Seikagaku Corporation, Japan); monoclonal rat-anti-mouse C3 (1:500) and monoclonal rat-anti-mouse C4 (1:1000, Cedarlane, Burlington, NC); polyclonal rabbit-anti-mouse C1q (1:500, generous gift from Dr. Andrea J Tenner, University of California, Irvine). Biotinylated donkey anti-rat IgG and donkey anti-rabbit IgG (both at 1:200, Vector Lab), followed by Fluor590-conjugated streptavidin (1:500, Invitrogen, Carlsbad, CA) and Fluor488-conjugated anti-mouse antibody (1:500, Invitrogen) were used for immuno-detection.

The brain sections were examined using an Olympus BX60 fluorescent microscope (Olympus America Inc., Center Valley, PA) with Olympus DP10 camera (Olympus) and ASI MS-2000 motorized stage (Applied Scientific Instruments, Eugene, OR). Pictures were taken with 10x eye-piece and 40x objective.

### Western blot

Micro-dissected tissue was removed from the thalamic (with hippocampus) and cortical (frontal cortex) brain regions of 12-months old Tg-SwDI and non-transgenic (C57BL/6) mice (from the other hemisphere of the same animals used for histology). Half of each group were homogenized on ice in lysis buffer containing 1% SDS, 0.5% IGPEL in PBS with Complete protease inhibitor (Roche, Basel, Switzerland) at 10 ml/g tissue ratio. The protein concentration was determined using the bichinchonic acid method (Pierce, Rockford, IL). Equal amounts of protein (50 μg) were loaded, fractionated on 10% SDS-polyacrylamide gels, and subsequently electrophoretically transferred to nitrocellulose membranes (Schleicher and Schuell, Hertogen-bosch, Netherlands) in blotting buffer (25 mM Tris-HCl, pH 8.6, 192 mM glycine, and 20% methanol). Blots were washed for 15 min in PBS containing 0.05% Tween 20 (PBST), pre-incubated with blocking solution (5% non-fat milk powder in PBST), washed three times with PBST, and subsequently incubated with primary antibodies and peroxidase-labeled secondary goat anti-rat or donkey anti-rabbit antibodies (Dako, Glostrup, Denmark). Detection was performed by chemiluminescence according to the description of the manufacturer (Boehringer Mannheim, Almere, Netherlands) and exposed to Kodak (Rochester, NY) X-OMAT-R films and quantified using VersaDoc imaging system (BioRad, Hercules, CA). The molecular mass of specific bands was determined by comparing to the BenchMark pre-stained protein ladder (Invitrogen) electrophoresed on the same gels.

### Real-time reverse transcriptase polymerase chain reaction

Total RNA was isolated from the tissue using RNeasy Lipid Tissue Kit (Qiagen, Hilden, Germany) as per manufacturer's instructions. Single stranded cDNA was prepared using 10 μl of total RNA using iScript cDNA Synthesis Kit (Biorad) per manufacturer's instructions. 6-carboxyfluorescein (FAM) 5' end labeled Taqman Assay on Demand primer and probe sets (Applied Biosystems, Foster City, CA) were used to assess expression levels of C1q (Mm00437836_m1) and C3 (Mm01232773_m1) for each cDNA sample. All samples were normalized against the endogenous control, TATA box binding protein (Mm00446973_m1). 500 ng of each cDNA sample was used in conjunction with 8 pmoles of each primer/probe set plus Taqman Universal Master Mix (Applied Biosystems). Each sample was analyzed in triplicates. Real-time quantitative PCR was performed on an Opticon2 (Biorad) using the following program: 95°C for 10 sec, followed by 40 cycles of denaturation, 95°C for 15 sec and anneal/elongation, 60°C for 1 min. The relative expression level of C1q or C3 was computed with respect to the mRNA expression of the endogenous control using the following formula: Relative mRNA expression = 2^-(-Ct gene of interest-Ct TATA)^

Ct is the threshold cycle value [22,23].

### Statistical analysis

Biochemical and molecular data were analyzed by ANOVA single factor test at α = 0.05 significance level.

## Results

### C1q, C3, and C4 were induced in Tg-SwDI mice

**Figure 1:**
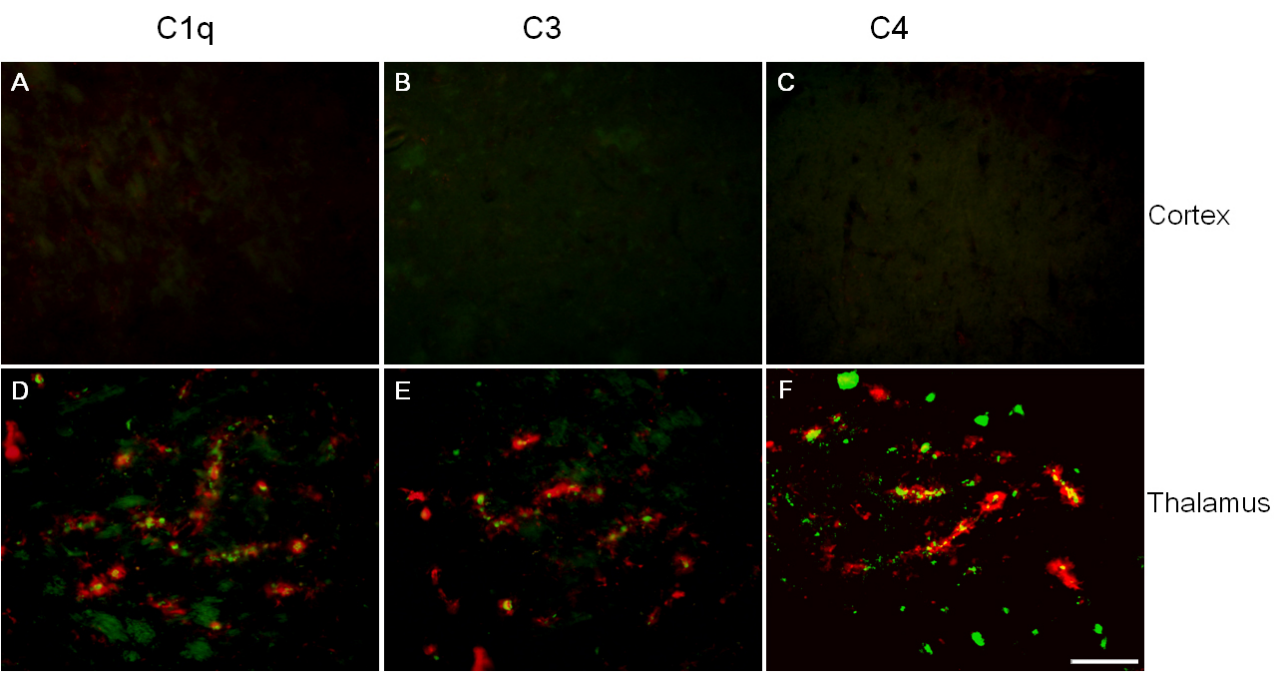
Immunoreactivities of native complement proteins C1q (A, D), C3 (B, E), and C4 (C, F) expressions were elevated in Tg-SwDI mice over wild-type. Forebrains of twelve months old Tg-SwDI and C57BL/6 mice were stained for fibrillar Aβ using thioflavin-S amyloid staining (green) and every one of the three complement proteins with specific antibodies (red). Positive complement immunoreactivity was associated with amyloid staining seen in thalamic regions (D, E, F), but not in cortical regions (A, B, C). Scale bars = 50 μm.

In our previous studies, we showed that in Tg-SwDI mice, thioflavin-S positive fibrillar Aβ deposits were primarily found to be associated with the microvasculature in the thalamus, subiculum, as well as hippocampus [9]. However, in the cortex Aβ deposits were largely in the parenchyma and in diffuse form. In the transgenic mice, activation of microglia and astrocytes was also elevated along with the microvascular fibrillar Aβ accumulation in the thalamic and hippocampal regions [10]. Since complement proteins have been reported to be present in reactive inflammatory cells associated with amyloid deposits, we first examined the presence of the three early native complement components in different brain regions of Tg-SwDI and wild-type C57BL/6 mice and their relationship with fibrillar Aβ deposits in the transgenic mice. Brains from twelve months old mice were harvested and processed as described in Materials and Methods. Immunostaining for C1q revealed very little cellular labeling in either the cortex of transgenic animals (Fig 1A) or the entire C57BL/6 wild-type mouse brain (data not shown). However, in thalamic region of Tg-SwDI mouse brains (Fig 1B), C1q was present in many cells associated with the microvascular fibrillar amyloid. Similarly, there were minimal immunoreactivities for either C3 or C4 in the cortical region of Tg-SwDI (Fig 1C and 1E, respectively) or any region of C57BL/6 mice (data not shown), but strong staining in the thalamus of Tg-SwDI mice (Fig 1D and 1F, respectively). Increased immunostaining of C1q, C3, and C4 was also detected in hippocampus and subiculum (data not shown), another two regions with prominent fibrillar Aβ deposition.

### Complement proteins are synthesized in Tg-SwDI mouse brain

**Figure 2:**
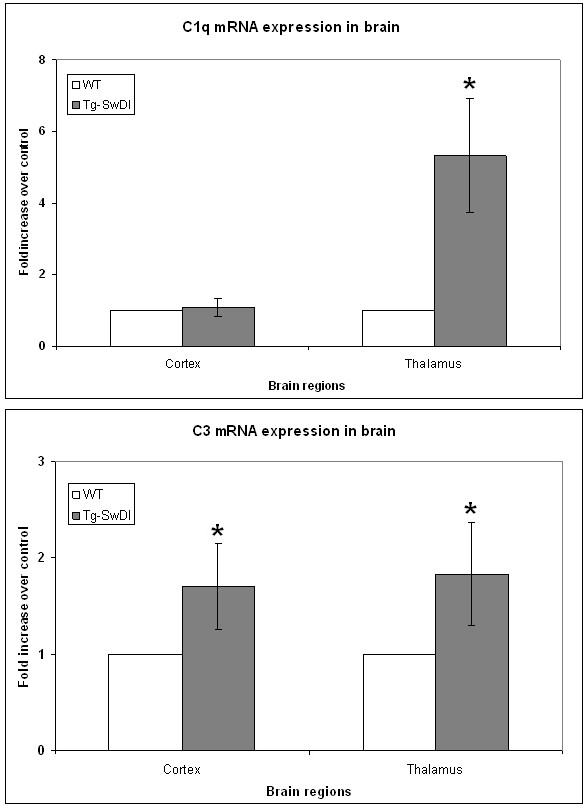
C1q and C3 mRNAs were increased in Tg-SwDI mouse brains. Real-time PCRs for C1q (A) and C3 (B) were performed on cDNAs synthesized from mRNAs extracted from twelve months old C57BL/6 (white bars) and Tg-SwDI (gray bars) mouse cortical and thalamic regions. Data shown are mean ± S.D. (n = 9). Both C1q and C3 were statistically significantly higher in transgenic mice over wild-type (*p < 0.02) except C1q in cortex.

**Figure 3:**
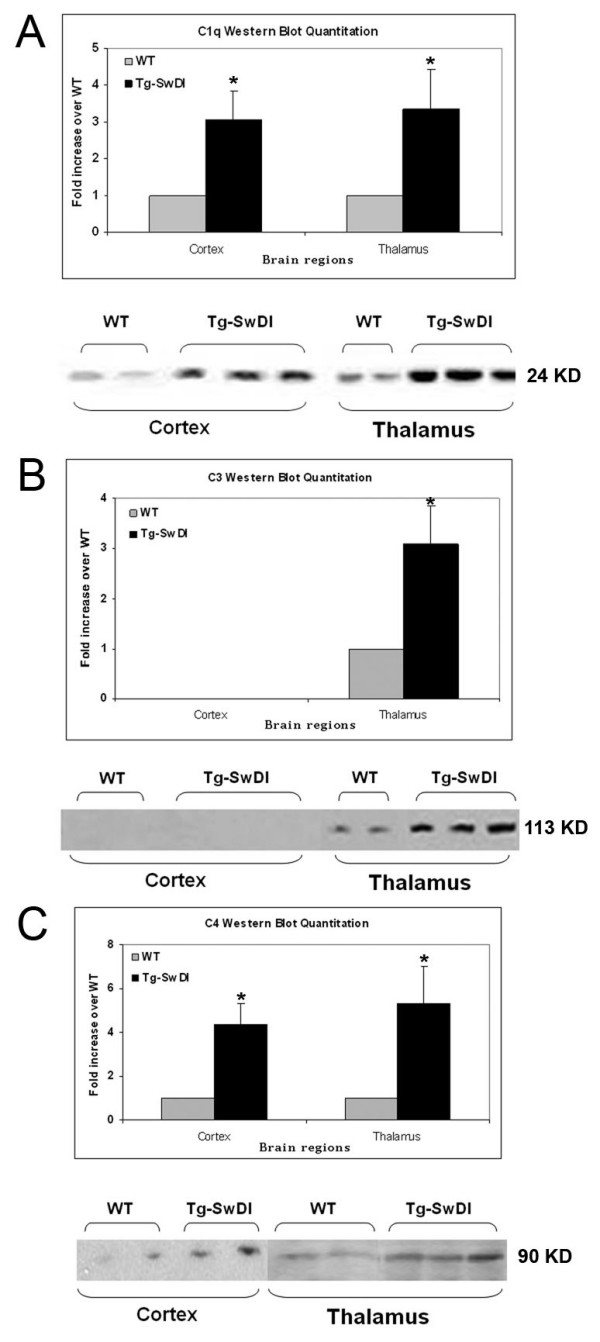
C1q, C3, and C4 protein levels are elevated in Tg-SwDI mice thalamic region. Western blot analysis of complement proteins was performed using rabbit anti-C1q (A), rat anti-C3 (B), and rat anti-C4 (C) antibodies. Data shown are mean ± S.D. (n ≥ 6). The increase of all three proteins in both regions of the transgenic over wild-type reached statistical significance (*p < 0.01) except C3 was undetectable in cortex. Representative western blots are shown below the quantitation.

Many studies have demonstrated that complement components can be generated locally in brain. Given that no prominent hemorrhage or compromised blood brain barrier has been observed in Tg-SwDI mice, the elevated expression of cerebral complement components is unlikely from peripheral leakage. To test this hypothesis, we next investigated the messenger RNA productions of these proteins to determine that the increase of complement expression was due to local synthesis. Quantitative real-time PCR was performed for C1q and C3 on mRNAs extracted from micro-dissected brain regions, using TATA box binding protein as the housekeeping gene. The data showed that there was a significant increase (p < 0.02) in the mRNA levels of both C1q and C3 in the thalamic regions of Tg-SwDI over wild-type (Fig 2). The increase in cortex of C3 mRNA was also statistically significant (p < 0.02), but not of C1q mRNA (Fig 2). To confirm that the increase in mRNA was translated into changes in protein levels, we performed quantitative Western blot analysis for these three complement proteins on homogenates from micro-dissected thalamic and cortical regions. In thalamic region, the results were consistent with both the immunohistochemical and real-time PCR data that protein levels of C1q (Fig 3A), C3 (Fig 3B), and C4 (Fig 3C) were statistically significantly higher (p < 0.01) in Tg-SwDI mice compared to C57BL/6 mice. In cortex, C1q and C4, but not C3 (undetectable in either genotype), were also significantly elevated (p < 0.01) in Tg-SwDI mice.

### Complement proteins are expressed in activated microglia of Tg-SwDI mice

**Figure 4:**
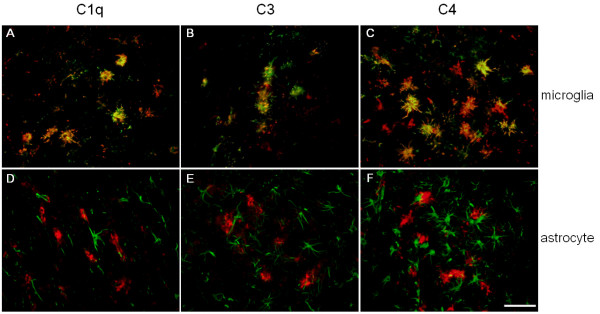
C1q, C3, and C4 are expressed in activated microglia not astrocytes. Colocalization of activated microglial cells (green A,B,C) or reactive astrocytes (green D,E,F) and complement components C1q (red A,D), C3 (red B,E), and C4 (red C,F) in the thalamic regions of twelve months old Tg-SwDI mice. Scale bars = 50 μm.

In Tg-SwDI mice, we previously reported that activated microglia and astrocytes were greatly enhanced in association with microvascular fibrillar Aβ, thus were most dense in the thalamic and hippocampal regions rich in these deposits. Both microglia and astrocytes, when activated, can produce many complement components. In order to investigate the cell types that were responsible for the induction of complement synthesis in Tg-SwDI mice, brain sections were immunolabeled for both complement proteins and markers of activated microglia or reactive astrocytes. The cells positive for complement proteins morphologically resembled activated microglial cells. When fluorescent images of both stainings were merged, it was clear that while almost no astrocytes were positive for any of the three complement proteins (Fig 4), most of the complement-positive cells were strongly labeled with antibody for activated microglia (Fig 4). Again, in hippocampus and subiculum, C1q, C3, and C4 immunoreactivities were also greatly overlapped with microglial cells, not astrocytes (data not shown).

## Discussion

We have reported before that in Tg-SwDI mice, in spite of the low expression level of transgene encoded human AβPP, profuse fibrillar amyloid deposits were present in cerebral microvessels, likely due to the highly fibrillogenic property of the Dutch/Iowa CAA mutant Aβ peptide, with no detectable export of this Aβ into the periphery [9]. In association with the microvascular fibrillar Aβ deposits, reactive microglia and astrocytes were found in these animals, and increased with age in parallel to the microvascular Aβ deposition [10]. We also detected pro-inflammatory cytokines such as TNFα, IL-1β, and IL-6 to be enhanced in Tg-SwDI mice, suggesting that these activated cells are actively expressing inflammatory molecules surrounding the microvasculature [10,11]. Previously, it has been shown that complement components are induced in AD patients and familial CAA patients as well as brains of AD animal models [24]. In addition to the well-studied pathogen elimination by complement activation, amyloid clearance may be promoted by C1q binding to fibrillar amyloid deposits, and generation of an opsonin, the C3b fragment [15,25]. Human studies have also shown increasing complement staining with growing presence of CAA [26]. However, due to the inefficient binding of mouse C1q to human Aβ, complement activation is much less in mouse models for AD than in AD patients [27], which might explain why we did not observe the presence of late-stage complement activation products. In brain, activated microglia are the major source of complement proteins in brain, although reactive astrocytes and neurons have also been shown to express complement when stimulated [28,29].

Here we demonstrate that early native classical complements C1q and C4, as well as C3, an important player in both classical and alternative complement pathways, are up-regulated in Tg-SwDI mouse brains. This induction was likely due to local synthesis since we have detected the increase in messenger RNA and protein levels by real-time PCR technique and Western blot, respectively. The observed differences between mRNA and protein levels of C1q and C3 were possibly due to several reasons. For example, increased levels of certain complement proteins may reflect decreased turnover and/or accumulation with amyloid deposits in the absence of increased expression. Also, the sensitivities of real-time PCR and Western blot are different depending on primers and antibodies, and the message levels may not be translated linearly to protein expression.

The immunoreactivities of C1q, C3, and C4 were cell associated and appeared to track along cerebral microvascular amyloid deposits, particularly in thalamus and subiculum (data not shown). We then investigated the cell type that expressed the complement. Double immunofluorescent labeling indicated that most complement-positive cells were activated microglia, not astrocytes. Although not all complement-positive cells overlapped with activated microglial labeling, this was possibly due to the multiple activation stages and types of microglia and one activation marker (keratan sulfate in this case [30, 31] may not reflect all activated microglial cells. These findings further support the notion that microglia in the Tg-SwDI mouse model are actively participating in the neuroinflammatory response and provide more insight into the specific reactions of microglia during disease progression as well as modulations of these reactions. In light of our recent work in which we detected improvement of memory performance in animals when microglial activation was suppressed [11], the effect of that inhibition on complement induction remains to be investigated to further elucidate the function of elevated complement in this model.

## Conclusion

In summary, complement proteins C1q, C3, and C4 were induced in Tg-SwDI mice in proximity to the cerebral microvasculature. This increase in expression was primarily in thalamus and hippocampus, and also in subiculum by immunohistochemistry (data not shown), regions that contain the highest amount of fibrillar microvascular amyloid deposition. These complement proteins were locally synthesized in brain, and mostly by activated microglial cells associated with microvascular Aβ deposits.

## Competing interests

The author(s) declare that they have no competing interests.

## Authors' contributions

RF processed the animal tissue, performed immunohistochemistry and Western blot, analyzed the data, and drafted the manuscript. KD performed the real-time PCR analysis. WVN contributed to the design of the study, guided data interpretation and presentation and edited the manuscript. All authors have read and approved the final manuscript.
